# Identification of *kakusei*, a Nuclear Non-Coding RNA, as an Immediate Early Gene from the Honeybee, and Its Application for Neuroethological Study

**DOI:** 10.3390/ijms131215496

**Published:** 2012-11-22

**Authors:** Taketoshi Kiya, Atsushi Ugajin, Takekazu Kunieda, Takeo Kubo

**Affiliations:** 1 Division of Life Sciences, Graduate School of Natural Science and Technology, Kanazawa University, Kakuma-machi, Kanazawa, Ishikawa 920-1192, Japan; 2 Department of Biological Sciences, Graduate School of Science, The University of Tokyo, Bunkyo-ku, Tokyo 113-0033, Japan; E-Mails: a-ugajin@biol.s.u-tokyo.ac.jp (A.U.); kunieda@biol.s.u-tokyo.ac.jp (T.K.); stkubo@biol.s.u-tokyo.ac.jp (T.K.)

**Keywords:** honeybee, *kakusei*, immediate early gene, insect, brain, non-coding RNA, foraging behavior, dance communication, Japanese honeybee, hot defensive bee ball formation

## Abstract

The honeybee is a social insect that exhibits various social behaviors. To elucidate the neural basis of honeybee behavior, we detected neural activity in freely-moving honeybee workers using an immediate early gene (IEG) that is expressed in a neural activity-dependent manner. In European honeybees (*Apis mellifera*), we identified a novel nuclear non-coding RNA, termed *kakusei*, as the first insect IEG, and revealed the neural activity pattern in foragers. In addition, we isolated a homologue of *kakusei*, termed *Acks*, from the Japanese honeybee (*Apis cerana*), and detected active neurons in workers fighting with the giant hornet.

## 1. Introduction

**Figure 1:**
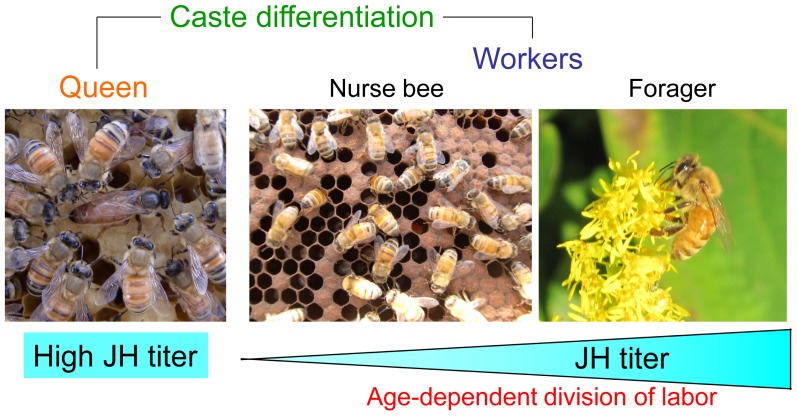
Honeybee society. Female adults are differentiated to reproductive queen and workers in the colony. A single queen lays 1000 eggs every day for reproduction, while the 30,000–50,000 workers are sterile and engage in tasks for colony maintenance. The workers change their tasks in an age-dependent manner (division of labor). Young workers engage in tasks inside the nest (nurse bees), while older workers engage in foraging (forager). Caste differentiation and task changes are regulated by juvenile hormone (JH).

The European honeybee (*Apis mellifera* L.) is a eusocial insect that organizes a highly sophisticated society (Figure 1) [1,2]. The honeybee colony comprises one queen, 30,000–50,000 workers, and 1000 drones, and the reproductive castes are clearly differentiated. The reproductive roles are held only by the queen and drones, and the social labors in the hive are managed only by the workers. Although the workers are female, they are sterile and engage in social labors such as brood rearing, nest building, guarding, and foraging. The labor of the workers changes depending on the age after eclosion [1,2]: young workers (less than two weeks old) work inside the hive as nurse bees, while older workers (more than two weeks old) work outside the hive as foragers. Some workers become guard bees that defend the hive entrance before they become foragers from nurse bees. The age-dependent division of labor is considered to be regulated by a sesquiterpenoid hormone, juvenile hormone (JH), whose titer increases in an age-dependent manner in workers [2,3]. Recent transcriptome analysis revealed that expression of a common set of genes change depending on the labor transition and exogenous JH application [4–6]. Despite identification of humoral factors and the completion of transcriptomic profiling, the neural mechanisms of social behaviors remain elusive.

**Figure 2:**
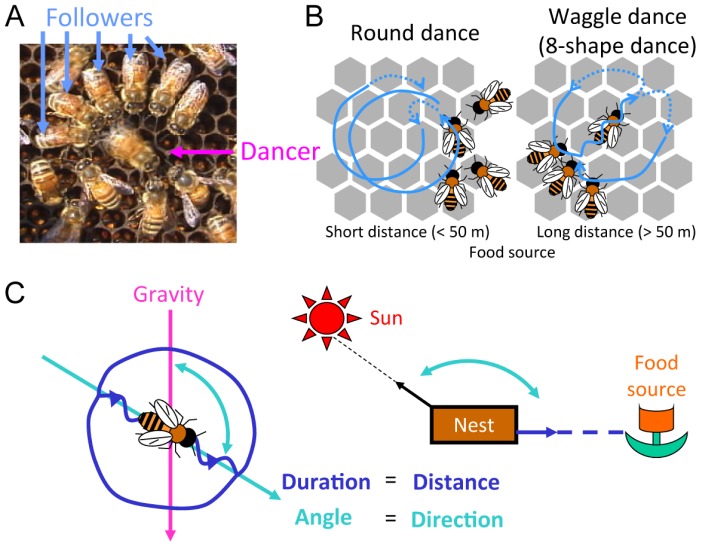
Dance communication of the honeybees. (**A**) A forager that finds a rich food source transmits the location of food source by dance communication (dancer: red arrow). Nestmates (followers: blue arrow) follow the dances several times, decode the dance information, and finally reach the indicated food source; (**B**) Dancers show different types of dance depending on the distance of the food source from the nest. When a food source is a short distance (<50 m) away, dancers perform round dances. In contrast, when a food source is a long distance (>50 m) away, dancers perform waggle (8-shape) dances; (**C**) In the waggle dance, foragers indicate the distance and direction to the food source by the duration and angle, respectively, of the waggle-phase of the dance. Foragers see the opposite direction of gravity as the sun direction. Thus, the dance is considered a miniature representation of the foraging trip.

One of the most interesting social behaviors of the honeybees is “dance communication” (Figure 2A), which was revealed by von Frisch, the Nobel Prize winner [7]. Foragers that successfully find a rich food source return to the hive and transmit the information regarding the location of the food source to their nestmates using a symbolic dance. They perform a “round dance” when the food source is a very short distance away from the hive (less than approximately 50 m) and a “waggle dance” when the food source is a long distance away (>50 m) (Figure 2B) [7,8]. Although with round dances, dancers transmit only the information that a good food source exists near the hive, foragers that follow the dances (followers) can locate the food source by utilizing the flower odor attached to the dancers. With waggle dances, dancers communicate the distance and direction of the food source from the hive by the duration and angle of the waggle-phase of the dances, respectively (Figure 2C). Thus, waggle dancers perform a miniature representation of the foraging trip by assuming that the direction opposite to gravity is the direction of the sun. Surprisingly, followers can decode the dance information into spatial information about the food sources, which allows them to access the indicated food source several kilometers away from the hive [7–9]. In the aspect that abstract information and concrete information are coded and decoded between individuals, dance communication of the honeybee is considered a symbolic communication. Symbolic communication, including human language, is considered to require a higher brain function and is found only in higher mammals, except for honeybees.

Recent studies revealed that foragers estimate flight distance using a visually driven odometer based on the amount of optic flow (the flow of visual information that crossed the visual field) that they perceive during the foraging flight [10,11]. On the other hand, foragers estimate the flight direction using the sun as a compass [7]. Thus, it is expected that foragers integrate this sensory information during foraging and encode it in the brain before they perform dances in the hive. Although there is a considerable amount of research concerning the sensory basis of these remarkable abilities [10–13], almost nothing has been known about the underlying neural mechanisms.

To elucidate the neural mechanisms of this remarkable ability, we first aimed to identify active brain regions in the foragers that might be involved in dance communication and/or information integration during the foraging flight. In the present paper, we review how we constructed neural activity mapping methods with the neural activity marker gene *kakusei*, a novel nuclear non-coding RNA [14], and the honeybee brain function that was revealed by our approach [15,16]. Next, we describe that *kakusei* has various transcript variants, which includes an inducible isoform and constitutive isoforms, and discuss the possible neural function of *kakusei [*17]. Third, we discuss the isolation of a homologue of *kakusei*, termed *Acks*, from the Japanese honeybee (*Apis cerana*), and detection of neurons that are active in the workers fighting with the giant hornet, the natural enemy of Japanese honeybees [18].

## 2. Identification of a Novel Non-Coding IEG, *kakusei*, which Can Be Used as a Marker to Visualize Neural Activity in the Honeybee Brain

**Figure 3:**
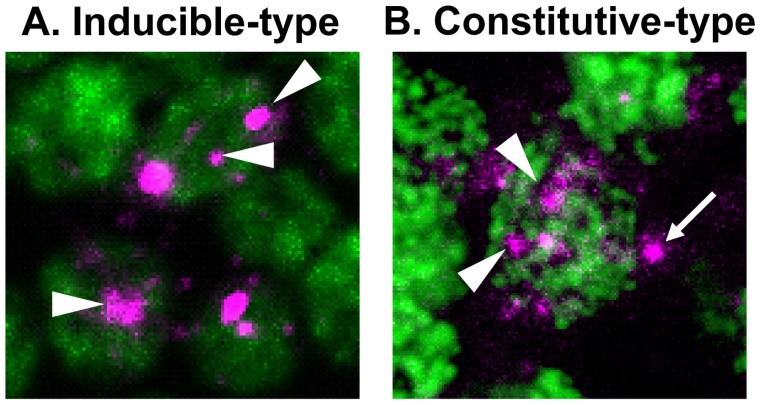
Distinct subcellular localization pattern of *kakusei* transcripts. (**A**) Inducible-type *kakusei* variant, which was initially isolated as a neural activity-induced non-coding RNA, localized exclusively in the nuclei (arrowheads: *kakusei* is shown in magenta, while nuclear DNA is shown in green); (**B**) Constitutive-type of *kakusei* variant, which was identified in a later analysis, localized mainly inside the nuclei (arrowheads) and partially in the periphery of the nuclei (arrow).

Immediate early genes (IEGs) are genes expressed in a neural activity-dependent manner and are widely used to visualize neural activity in various vertebrates’ species [19–24]. IEG mapping methods are advantageous for identifying active neurons in free-moving animals at the cellular level, although the time resolution is not good (IEGs detect the summation of neural activity over 30 to 60 min). Because foragers fly a long distance and move rapidly, it is practically impossible to detect neural activity with conventional electrophysiology and calcium imaging methods. Thus, we examined the neural activity pattern in the forager brain using an IEG mapping approach. At the start of our studies, despite the wide application of IEG mapping in vertebrates, no IEG had yet been identified in insect brains and thus we began our study by identifying IEGs from honeybee brains. Using the differential display method, we successfully identified a novel IEG, termed *kakusei* (“awakening” in Japanese), whose expression is strongly induced by seizures that can be induced by awakening workers from anesthesia [14]. This was the first identification of an IEG from the insect brain. Although many of the IEGs in vertebrates encode transcription factors that regulate downstream gene expression, there was no significant open reading frame among any of the possible reading frames of the *kakusei* cDNA sequence (approximately 7 kb long) and *kakusei* transcripts localized exclusively in the neuron nuclei, suggesting that the *kakusei* transcript functions as a non-coding RNA (Figure 3A). Although some microRNAs that are expressed in response to neural activity are reported in vertebrates [25,26], no long non-coding RNA that is expressed in a neural activity-dependent manner had been identified. Thus, *kakusei* is the first example of a long non-coding nuclear RNA that shows an immediate early response to neural activity.

**Figure 4:**
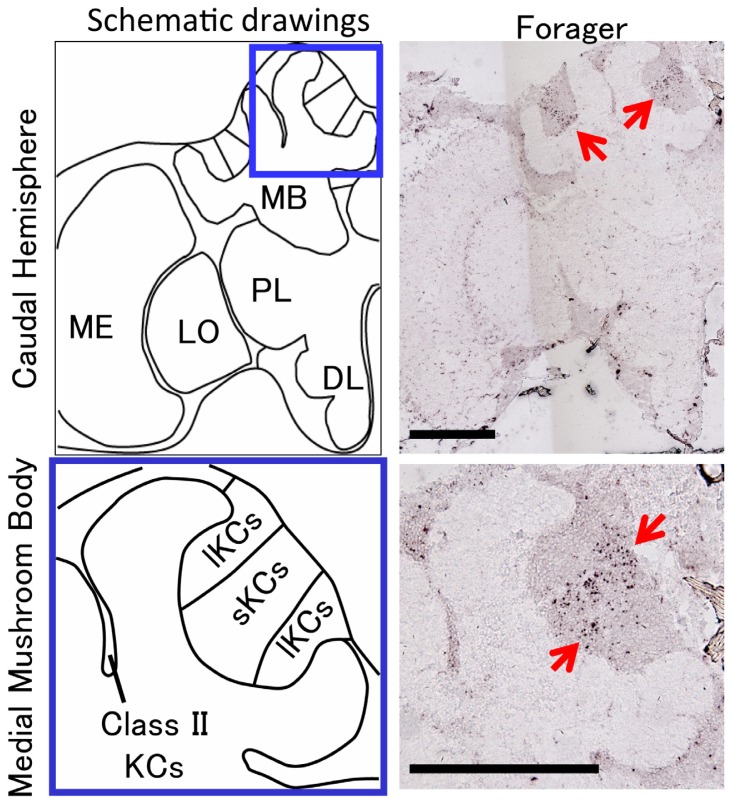
Pattern of *kakusei* expression in the forager brain. Left panels are schematic drawings of a brain hemisphere (upper panel) and mushroom bodies (lower panel) of the honeybee worker. Right panels are pictures of *in situ* hybridization of *kakusei* in the forager brain. As indicated by the red arrows, *kakusei* was preferentially expressed in the small-type Kenyon cells. Abbreviations: DL, dorsal lobe; MB, mushroom body; ME, medulla; LO, lobula; PL, protocerebrum lobe; lKCs, large-type Kenyon cells; sKCs, small-type Kenyon cells; class II KCs, class II Kenyon cells. Bars indicate 250 μm.

To visualize the neural activity pattern in the forager brain, we established high-sensitivity *in situ* hybridization methods that allowed us to detect neural activity patterns from the entire brain. Surprisingly, forager brains showed a characteristic *kakusei* expression pattern. The *kakusei* expression was preferentially detected in a subset of mushroom body (MB) neurons (Figure 4). The Kenyon cells (KCs) of honeybee MBs are classified into two distinct types, termed class I and class II KCs. Whereas somata of the class I KCs are located inside of the MB calyces, somata of the class II KCs are located outside of the calyces. The class I KCs are further subdivided into two types of intrinsic neurons, termed large-type Kenyon cells (lKCs) and small-type KCs (sKCs), based on their cell body sizes [27–30]. The sKCs locate in the inner core of each calyx and the lKCs locate in the inside edge of each calyx, respectively (Figure 4). The most prominent *kakusei* expression was observed in the sKCs, whose somata are located in the center of the MBs. In addition to the sKCs, moderate *kakusei* expression was observed in neurons of the optic lobes, which are the visual center of the insect brain, consistent with the notion that foragers utilize visual information during their foraging trips. Based on a thorough comparison of behavior and *kakusei* expression, we concluded that preferential neural activity in the sKCs is closely related to foraging behavior, but not to other stimuli/behaviors such as visual stimulation, flying behavior, or orientation behavior. Further, we revealed that the level of *kakusei* expression in the MB neurons was closely related to the number of foraging trips [15,16]. These findings strongly suggest that sKC-preferential neural activity is associated with behavioral components that are specific to foraging behavior. Our studies are the first to identify the active brain region that is related to foraging behavior of honeybees, and also might be involved in dance communication. The MBs are important brain sites for higher sensory integration like learning and memory in the insect [31–33]. In the honeybee brain, all sensory modalities investigated (visual, olfactory, gustatory, and mechanosensory) project to the MBs, suggesting that multimodal sensory information is integrated in the MB neural circuits [27,28,34–38]. Although the inputs and outputs of the sKCs are well investigated, the role of the neural circuitry composed by the sKCs has been unknown [27,28,34,35,38]. Our studies shed light on the importance of sKC function for foraging behavior and partially elucidated a functional subdivision of the MB neurons under natural behavioral conditions. We expect that future research will elucidate the functional importance of the sKCs for foraging behaviors.

## 3. Identification of Constitutive-Type Transcript Variants Implicates Cellular Function of *kakusei*

In the process of investigating the transcriptional unit of *kakusei*, we observed that *kakusei* has several transcript variants. With Northern blot and reverse transcription-polymerase chain reaction analyses, we revealed that, in addition to neural activity-inducible *kakusei* transcripts (inducible-type variant), multiple variants are constitutively expressed in the honeybee brains independent of neural activity (constitutive-type variant) [17]. Furthermore, the constitutive-type variants are expressed in neurons throughout the entire brain and are localized predominantly inside the neural nuclei and partly in the periphery (Figure 3B), indicating that the spatial distribution pattern is distinct between the inducible-type and constitutive-type variants. In our analysis, there was no evidence of alternative splicing within the *kakusei* locus or extended transcription from the *kakusei* cDNA sequence. Therefore, we assume that the constitutive-type variants are produced by differential transcription initiation or termination.

What do these distinct variants suggest about the cellular function of *kakusei*? In mammals, neural activity-dependent induction of variant transcripts is reported for IEGs like *bdnf* and *homer*[39–41]. Activity-dependent transcription from alternative promoters or splicing occurs in these IEGs. In *homer*, activity-dependent products regulate synaptic function through competition with constitutive products [42,43]. In addition, in nuclear-retained *mCAT2* mRNA, stress-induced posttranscriptional cleavage of the 3′ untranslated region is known to produce transcript variants [44], which results in a rapid response to cellular stress. Consistent with these previous studies, we hypothesize that the constitutive-type transcripts have a specific cellular function under a dormant state and neural activity-induced expression of the inducible-type variants affects function in a cooperative or competitive manner. The nucleus in higher eukaryotes consists of distinct domains, including the nucleolus, speckles, paraspeckles, and Cajal bodies [44,45]. In these domains, chromosomes are less condensed, and specific marker proteins and particular groups of RNA molecules are concentrated [44–46]. These domains have various roles in RNA metabolism within the nucleus, such as splicing, processing, and RNA editing [45]. Our subcellular localization of *kakusei* variants revealed that each variant localizes to some nuclear subdomain, where chromosomes are less condensed and DNA staining is weak (Figure 3A,B). Therefore, we speculate that *kakusei* is involved in RNA metabolism in the nuclei, which is regulated in a neural activity-dependent manner, although further studies are necessary to reveal the cellular function of *kakusei*.

## 4. Isolation of *Acks*, a Homologue of *kakusei* from the Japanese Honeybee, and Detection of Active Neurons in the Workers Fighting with the Giant Hornet

**Figure 5:**
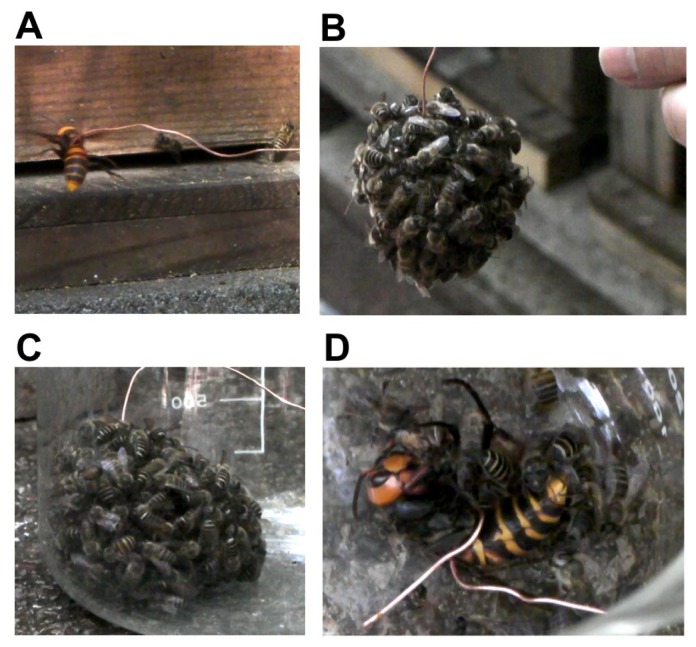
Artificial hot defensive bee ball formation. A hot defensive bee ball is usually formed in the beehive. To collect only the workers involved in forming the bee ball, we attached a giant hornet to the tip of a wire and introduced it into the hive (**A**) to allow the Japanese honeybees to form a bee ball around the giant hornet (**B**), and recovered the bee ball in a glass beaker (**C**). The decoy hornet inside the bee ball was dead 60 min after the formation of the bee ball (**D**).

One of the major challenges of neuroethological studies is to understand how adaptive behavior is acquired and evolved. As a good example of evolution of adaptive behavior, we focused on thermal defense behavior of the Japanese honeybee (*Apis cerana japonica*) [47,48]. In general, honeybees use their stingers to counterattack an intruder [2], but Japanese honeybees exhibit a distinct defensive behavior, called “hot defensive bee ball formation”, when they are attacked by the giant hornet (*Vespa mandarinia japonica*) [48]. In Japan, giant hornets are the most formidable natural enemy of the honeybees and they continuously attack honeybee colonies to steal their larvae and pupae in autumn [18,48,49]. Because the exoskeleton of the giant hornets is so rigid that bee stings are ineffective, colonies of European honeybees are often destroyed. The giant hornets inhabit only East Asia, including Japan, and the European honeybees were introduced to Japan in the Meiji era (about 140 years ago) for apiculture. Therefore, European honeybees do not have the ability to counterattack the giant hornets. In contrast, Japanese honeybees, which have long survived the continuous threat of predation by giant hornets, acquired and evolved this Japanese honeybee-specific defensive behavior [48]. When colonies of Japanese honeybees are attacked by giant hornets, a group of more than 500 workers quickly forms a spherical assemblage called a “hot defensive bee ball”, trapping the hornet inside the ball. In the ball, honeybees vibrate their flight muscles and produce heat. The temperature in the ball quickly rises to almost 47 °C, which is lethal to the hornet but not to the honeybees. The high temperature phase continues for approximately 20 min. Within approximately 30 to 60 min after initiating the bee ball formation, the hornet is killed by the heat [48,50] (Figure 5A–D). This anti-predator behavior is a good example that selective pressure to avoid predation resulted in species-specific behavioral evolution [47]. To gain insights into how this behavior is acquired and evolved, as a first step, we initiated studies to elucidate the neural basis of this thermal defensive behavior by IEG mapping [18].

**Figure 6:**
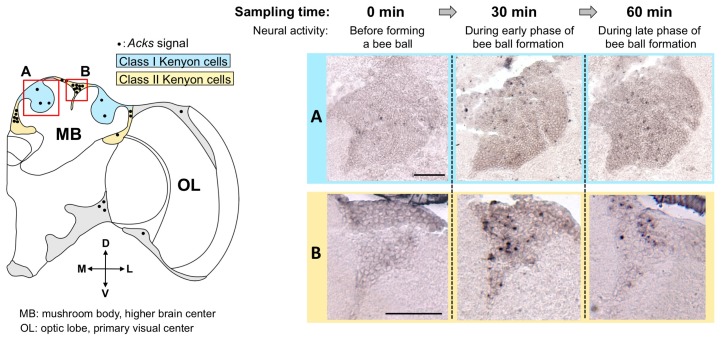
Active brain regions during the formation of a hot defensive bee ball. (Left panel) Schematic drawing of the honeybee brain hemisphere. *Acks* signals detected in the brains of workers collected 30 and 60 min after bee ball formation are schematically indicated with black dots. (Right panels) The results of *in situ* hybridization are shown in panels A and B, which correspond to the red boxes in the left panel. Many dotted *Acks* signals were detected at 30 (middle column) and 60 min (right column) after the bee ball formation. In contrast, minimal signal was detected at 0 min (left column). Note that the *Acks* signals were much denser in the Class II Kenyon cells (panels B). Bars indicate 100 μm.

First, we constructed a neural activity detection system using the IEGs. No *kakusei* homologue was found in other species whose genomes were determined, suggesting that *kakusei* is a honeybee-specific noncoding RNA. We were, however, able to isolate a homologous gene from the Japanese honeybee, termed *Acks* (*Apis cerana kaku*s*ei*), with comprehensive reverse transcription-polymerase chain reaction experiments [18]. Like *kakusei*, *Acks* is rapidly expressed in a neural activity-dependent manner and the transcripts are localized exclusively in the nuclei. These results suggest that *kakusei* is a conserved gene among the *Apis* genus and its expression property as an IEG is also conserved. With neural activity mapping by *Acks*, we found that class II KCs are preferentially active in the Japanese honeybee workers forming a hot defensive bee ball [18] (Figure 6). The MB structure of the Japanese honeybee is similar to that of the European honeybees, which comprises class I and class II KCs (Figures 4 and 6). Because *Acks* detects the summation of neural activity over 30 to 60 min, we next dissected behavioral components that might be involved in the bee ball formation and investigated *Acks* expression. We found that a similar *Acks* expression pattern (class II preferential expression pattern) is observed in the Japanese honeybee workers when they are exposed to a high temperature (46 °C). Further experiments with quantitative analysis revealed that half of the *Acks* expression remained even after we removed the antennae, which function as a temperature sensor in insects, suggesting that the bee brain directly senses the temperature as an internal thermal sensor. Based on recent findings in *Drosophila* that some transient receptor potential (TRP) channels are expressed in the brain and assumed to function as internal thermal sensors [51–60], we speculate that a similar mechanism is working in the brains of Japanese honeybee workers. Because *Acks* is preferentially expressed in the class II KCs of the fighting workers and heat-exposed workers, we also speculate that class II KCs functions for thermal sensing to appropriately regulate heat generation during forming the bee ball. The Japanese honeybee workers fight against the giant hornet by utilizing the small (3–5 °C) difference in lethal temperature between them (the lethal temperature of the giant hornet is approximately 45 °C, while that of the Japanese honeybee workers is 49 °C) [48,50]. Therefore, precise temperature monitoring and control of heat production is vital to Japanese honeybee workers. We expect that future studies will elucidate how accurate temperature control is attained in the Japanese honeybee worker brains.

## 5. Conclusions and Perspectives

In the present paper, we reviewed our recent studies using the IEG *kakusei* as a neural activity marker. In contrast to conventional IEGs, *kakusei* does not encode a protein and is suggested to function as a non-coding RNA in the nuclei. This exceptional property of *kakusei*, whose inducible isoform localizes exclusively in the nuclei, provided us a chance to quantitatively analyze *kakusei* expression, by counting the number of *kakusei*-positive cells. With this powerful approach, we successfully revealed some of the honeybee brain functions: foraging behavior of the European honeybees [14–16,61] and defense behavior of the Japanese honeybees [18]. Surprisingly, both behaviors induced behavior-specific neural activity patterns in the MBs. Although the MBs are well-studied as strong candidate brain regions for higher brain function in insects [32,33,62], our studies are the first to reveal how the MB neurons function in natural and adaptive behaviors. In these studies, however, we only observed the neural activity pattern of free-moving insects, and it is necessary to reveal cause-effect relationships between neural activity and behaviors in future studies. Recent progress in genome editing methods utilizing ZFN (Zinc-Finger Nuclease) and TALEN (Transcription Activator-Like Effector Nucleases) provide us with a chance to analyze gene function using a non-transgenic approach and theoretically make it possible to produce any gene-modified organism [63–65]. By applying these techniques to honeybees, we can knock-out *kakusei* function and manipulate neural activity by exogenously introducing optogenetic tools such as channelrhodopsin [66]. Although there is as yet no report for gene knock-out or transgenic honeybees, we expect that higher brain function and the neural basis of social behaviors, which are the prominent characteristics of honeybees, can be revealed in future studies using these novel techniques.
